# Enhancing reproducibility using interprofessional team best practices

**DOI:** 10.1017/cts.2020.512

**Published:** 2020-07-16

**Authors:** Betsy Rolland, Elizabeth S. Burnside, Corrine I. Voils, Manish N. Shah, Allan R. Brasier

**Affiliations:** 1 Institute for Clinical and Translational Research, School of Medicine and Public Health, University of Wisconsin-Madison, Madison, WI, USA; 2 Carbone Cancer Center, School of Medicine and Public Health, University of Wisconsin-Madison, Madison, WI, USA; 3 Radiology, School of Medicine and Public Health, University of Wisconsin-Madison, Madison, WI, USA; 4 Surgery, School of Medicine and Public Health, University of Wisconsin-Madison, Madison, WI, USA; 5 William S. Middleton Memorial Veterans Hospital, Madison, WI, USA; 6 Emergency Medicine and Internal Medicine, School of Medicine and Public Health, University of Wisconsin-Madison, Madison, WI, USA; 7 Internal Medicine, School of Medicine and Public Health, University of Wisconsin-Madison, Madison, WI, USA

**Keywords:** Team science, translational teams, reproducibility, ethics, interprofessional teams

## Abstract

The pervasive problem of irreproducibility of preclinical research represents a substantial threat to the translation of CTSA-generated health interventions. Key stakeholders in the research process have proposed solutions to this challenge to encourage research practices that improve reproducibility. However, these proposals have had minimal impact, because they either 1. take place too late in the research process, 2. focus exclusively on the *products* of research instead of the *processes* of research, and/or 3. fail to take into account the driving incentives in the research enterprise. Because so much clinical and translational science is team-based, CTSA hubs have a unique opportunity to leverage Science of Team Science research to implement and support innovative, evidence-based, team-focused, reproducibility-enhancing activities at a project’s start, and across its evolution. Here, we describe the impact of irreproducibility on clinical and translational science, review its origins, and then describe stakeholders’ efforts to impact reproducibility, and why those efforts may not have the desired effect. Based on team-science best practices and principles of scientific integrity, we then propose ways for Translational Teams to build reproducible behaviors. We end with suggestions for how CTSAs can leverage *team-based* best practices and identify observable behaviors that indicate a culture of reproducible research.

## Introduction

### The Problem

Funders like the National Institutes of Health and journal editors have invested substantial effort into enhancing the rigor of clinical studies by requiring, for example, pre-registration of clinical trials at ClinicalTrials.gov and publication checklists such as CONSORT. Yet, relatively little has been done to enhance the rigor of the preclinical research that is the foundation for new innovations, diagnostics, and therapeutics to be brought to the patient. If preclinical studies are not reproducible, downstream clinical trials designed to test or build upon these findings are destined to fail. Estimates of the frequency of irreproducible experiments in peer-reviewed research range from 75% to 90% [1]. Studies by industrial partners report that 75% of preclinical studies by academic scientists cannot be reproduced by qualified industry investigators, leading to delays, premature termination [2], and a loss of trust in academic research. In the US alone, cumulative costs of irreproducible preclinical research exceed 50% of research expenditures, resulting in the economic impact of ~$28B annually [3]. Consequently, preclinical work is poorly predictive of successful clinical outcomes, and the majority of drug failures are in phase II for lack of efficacy [4]. These challenges are compounded by a related – yet separate – problem of “inaccessible” studies, stemming from incomplete methodological reporting and lack of transparent data sharing. Together, these problems lead to estimates that 85% of biomedical research is “wasted” in terms of time, effort, and impact in that it never impacts human health [5].

### Dimensions of Reproducible Science

We embrace the definition proposed by a 2016 Perspective article in *Science* that defined three types of reproducibility: 1. methods reproducibility, 2. results reproducibility, and 3. inferential reproducibility [6]. Methods reproducibility refers to the “ability to implement, as exactly as possible, the experimental and computational procedures, with the same data and tools, to obtain the same results.” Results reproducibility focuses on the “production of corroborating results in a new study, having followed the same experimental methods.” Inferential reproducibility is “making the knowledge claims of similar strength from a study replication or reanalysis.” A comprehensive solution to the irreproducibility problem requires attention to and improvement of each type of reproducibility.

### Origins of Irreproducibility

There are many root causes of irreproducibility, including misconduct [fabrication, falsification, plagiarism, and misrepresentation (FFPM)] and Detrimental Research Practices (DRPs). DRPs include inappropriate authorship practices (honorary authorship), not retaining research materials (data, analysis code), neglectful supervision, misleading statistical analyses, deficient institutional compliance, and irresponsible publication practices by editors or peer reviewers [7]. A systematic review found that 1.97% of scientists admitted to engaging in FFPM, while 9.5% admitted to engaging in DRPs, making DRPs the more pervasive and larger problem [8]. Both of these numbers relying on self-report are likely underestimates. The 2017 National Academies of Sciences report, “Fostering Integrity in Research,” noted that DRPs can be reduced by adhering to six values of scientific integrity: 1. Objectivity, conducting one’s research while minimizing the impact of personal biases; 2. Honesty, reporting methods and data accurately; 3. Openness, being transparent about how the science was conducted; 4. Accountability, taking responsibility for and standing behind one’s work; 5. Fairness, judging others’ work on its own merits or allocating credit appropriately; and 6. Stewardship, giving back to the community [7]. Values, however, are notoriously refractory to training and challenging to measure. To impact reproducibility, they must be codified as explicit behaviors.

There have been increasing calls for research institutions to take an active role in this codification by operationalizing better research behaviors and improving local culture and climate through providing education on research integrity, such as responsible conduct of research (RCR) [7]. However, systematic reviews indicate that RCR training alone will not result in substantial improvement [9]. Furthermore, even if we succeed in impacting the behaviors and beliefs of individuals, environmental pressures and institutional incentives may inadvertently promote DRPs. For example, investigators with substantial citation impact garnered by publishing in high-impact journals are highly valued by institutions, promoted more easily and sometimes more rapidly, and their projects are more competitive for extramural funding [10]. An anonymous survey found that 20% of graduate students had been pressured to publish uncertain findings [10, 11], indicating institutional norms, power dynamics among collaborators, local culture, and incentives have a substantial impact on investigator behavior and may override any values-based training. Interventions that move beyond the local culture at an institution are being implemented by funders [12] and publishers [13], including emphasis by funders on data management plans and manuscript checklists from publishers. Reproducibility initiatives to independently replicate key studies are also being encouraged [14]. However, there is little evidence that these behaviors increase reproducible science.

### Too Little, Too Late

We contend that these proposals and interventions have had minimal impact on increasing reproducibility thus far, because they either 1. take place too late in the research process to impact its trajectory, 2. they focus exclusively on the *products* of research instead of the *processes* of research, and/or 3. they fail to take into account the driving incentives in the research enterprise. First, requirements by publishers such as submission checklists and independent statistical review come at the completion of the study, at which point it is too late to impact how the research was conducted. Funders require data sharing by large projects, but compliance is poor. Consequently, NIH expanded its data sharing policy to apply to all funded projects [15]. Yet, after the project has ended, there is little a funder can do to fix systemic data problems and ensure a data set worth sharing. Second, grant reviewers reward the high impact and innovation of research results such as publications or patents, without considering whether a project’s methods or results are reproducible. Although examining the scientific premise in NIH grant applications is an important step forward, transparency and durability of the findings are not independently scored evaluation criteria. Consequently, high-impact irreproducible scientific findings are still rewarded. Third, stakeholder interventions to enhance reproducibility may conflict or be meaningless if they do not take into account the complexity of the local and broader contexts. Journals encouraging replication studies will not encourage reproducibility if resulting manuscripts are low impact, with lower citation, funding agencies will not provide funding, and universities will not give faculty credit for conducting them.

### One Solution: A Focus on Translational Teams

How, then, do we incentivize and support reproducibility from the beginning of a project, focusing on the processes of research to ensure that the products are reproducible, while taking into account the competing incentives of the research environment? In the realm of Clinical and Translational Science, Translational Teams present one potential solution. As discussed, the challenges of irreproducible research are especially pronounced in the area of clinical and translational science, which is defined by its goal of translating basic preclinical research into patient care and, eventually, population–health impact. Furthermore, this work is primarily done in interdisciplinary teams, requiring different expertise as the science moves across the translational spectrum. The Clinical and Translational Science Awards (CTSAs) were developed, in part, to address this difficulty in translation and can be part of the reproducibility solution. CTSA hubs employ two key strategies to support bidirectional movement across the translational spectrum: *creating infrastructure* and *developing new, innovative approaches*. By focusing these two strategies on the team-based nature of clinical and translational science, the CTSA hubs have a unique opportunity to impact the culture of the research enterprise and, thus, increase the conduct of reproducible research. They can do so by focusing on introducing and supporting innovative, interprofessional team-based reproducibility practices.

Consistent with earlier work [16, 17], the 2019 CTSA Methods and Processes Domain Task Force working group “Developing Translational Science Team Competencies” endorsed the definition that the Translational Team is a special case of cross-disciplinary team formed to address unmet health needs. A Translational Team is composed of a dynamic and diverse membership that interacts, adapts, and evolves to address a shared translational objective; namely, to advance a product (device/drug/diagnostic), behavioral intervention, or evidence-based approach toward sustainable improvements in human health. It may work in one or more phases of translation, in a bi-directional manner, including preclinical, clinical, implementation, and population-based research. The Translational Team engages diverse disciplines and professions in generating new generalizable knowledge and training learning communities.

Perhaps most importantly, for the purposes of this discussion, Translational Teams are comprised of individuals working in complex research environments, full of competing incentives, and they interact in some way with the stakeholders discussed (funders, editors) who have an interest in increasing reproducible research. As such, they offer an opportune target for a “right-sized” intervention with a broader and deeper impact than training individuals, but which is still smaller and less complex than trying to change a university. Such an intervention, focused on building team-based reproducibility behaviors from the beginning, targeting the process of research, and taking into account competing incentives, can create an environment within the team that supports all three types of reproducibility. This reproducibility-supporting team environment can potentially counteract negative influences in the broader research environment. This focus creates a culture that supports and continuously reinforces reproducibility.

Over the past decade, the field of the Science of Team Science (SciTS) has identified team-based behaviors that increase productivity and scientific impact in teams. Many of these behaviors impact the reproducibility of the research, as described below. We contend that those team-based behaviors, when integrated with the values of scientific integrity and Good Institutional Practices (GIPs), can move the needle on reproducibility. Proposed by Begley et al. [10], GIPs include 1. discussion of research methods, 2. reporting systems, 3. training and standards, 4. records and quality management, 5. appropriate incentive/evaluation systems, and 6. enforcement. Instead of targeting the institutional level, GIPs can, instead, be operationalized and implemented at the team level, weaving both these practices and specific, measurable behaviors tied to scientific integrity, into behaviors that have been shown to impact team productivity. By integrating evidence-based team behaviors with reified values of scientific integrity and reproducibility-enhancing behaviors, we can create conditions in which reproducible science is not only more likely, it is actually easier to conduct than irreproducible science. CTSA hubs provide an ideal environment in which to develop the infrastructure and innovative approaches that create those conditions.

### Four Phases of Translational Team Evolution

**Fig. 1.**
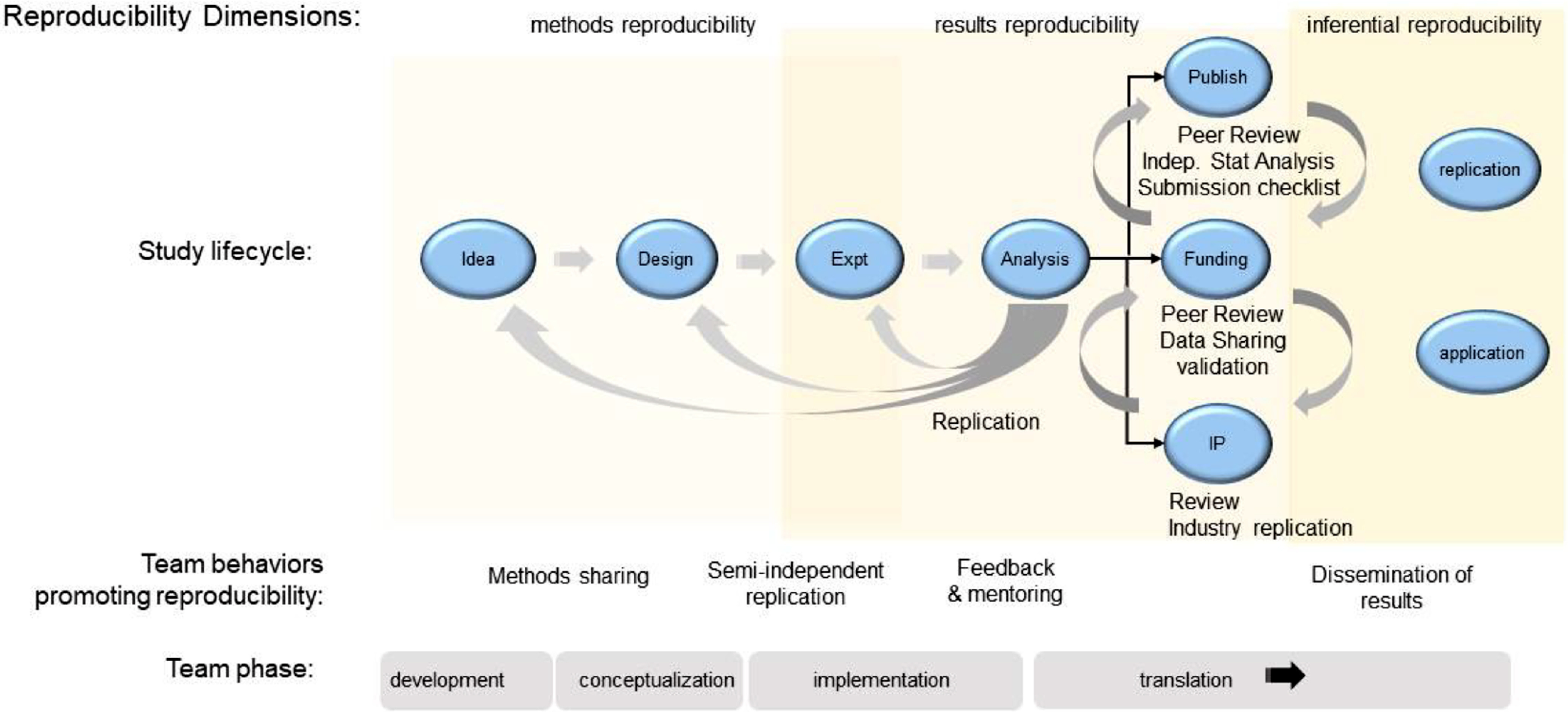
Lifecycle of a preclinical research project. Shown is a schematic view of the lifecycle of a preclinical research project related to the four developmental phases of a translational team. Behaviors for each reproducibility domain are indicated. Team behaviors that reinforce good institutional practices of reproducible science are indicated.

Interprofessional Translational Teams are dynamic and evolve through four distinct phases [18], illustrated in Fig. 1: development, conceptualization, implementation, and translation. In the Development phase, successful teams develop the problem space in which they plan to work, engaging in conversations to understand how their fields and perspectives fit together. This work includes developing a shared mission and goals, learning about one another’s scientific frameworks and methods, and developing a shared vocabulary. The work done during this phase sets the stage for how the team will interact as the project progresses, so it is critical that the team builds a culture of trust, openness, and inclusivity.

In the second phase, Conceptualization, the team dives more deeply into the research questions it will address, bringing together the viewpoints, methods, and approaches from the various disciplines required to answer those questions. The team must continue to have these cross-disciplinary conversations and hone their shared mental models and shared framework.

The Implementation phase is when the team will “launch, conduct, and refine” their research. It can be especially challenging for teams, because it requires iterative, ongoing discussions of the work being done by individuals, sub-teams, and the team as a whole, to ensure all activities are in service of the larger mission of the project. Shared protocols used to guide semi-independent replication may be necessary to achieve the project’s goals.

Finally, the Translation phase engages the team in applying their findings to the real-world problem. Each phase represents an opportunity for implementing SciTS best practices, scientific integrity behaviors, and GIPs, building in reproducibility from the beginning, focusing on the processes of research, and accounting for competing incentives while increasing all three types of reproducibility.

### Applying SciTS Best Practices

SciTS Researchers have identified behaviors of effective teams [19]. These behaviors create environments that support reproducible research, embrace the values of scientific integrity, and drive GIPs. Here, we focus on six SciTS best practices: 1. Develop a shared mission, vision, and goals; 2. Build a culture of trust, accountability, openness, inclusivity, and constant learning; 3. Facilitate interdisciplinary conversations on approaches, methods, and results; 4. Build strong research support systems; 5. Build accessible, transparent data management systems; and 6. Foster strong, functional leadership.

**Fig. 2.**
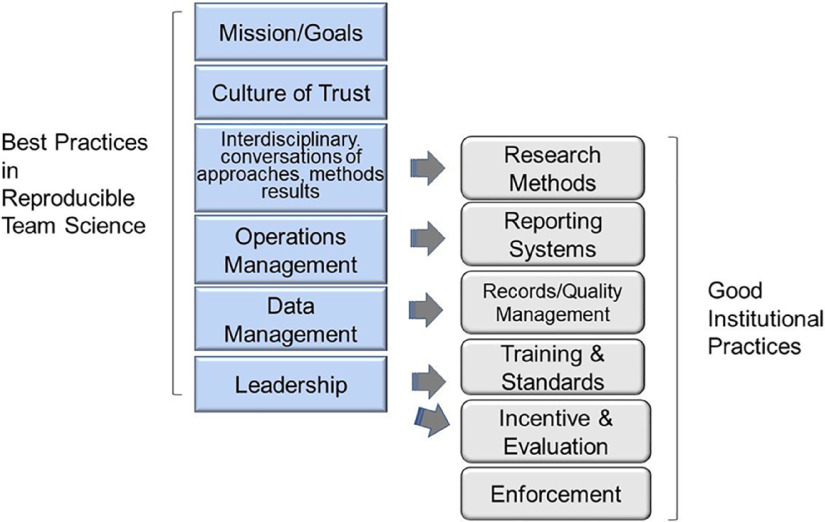
Best practices in reproducible team science support the culture of good institutional practices (GIPs). Mapping the best practices in reproducible team science with the six GIPs proposed by Begley et al.

These best practices occur throughout the four phases of Translational Team evolution and are self-reinforcing. For example, creating a culture of openness and inclusivity is both required by, and benefits from, interdisciplinary conversations. Both need to be addressed early in team formation and be ongoing concerns. Moreover, these behaviors seamlessly support five of the six GIPs proposed by Begley et al. [10], as illustrated in Fig. 2.

1. Develop a shared mission, vision, and goals. Shared mission, vision, and goals are highly related to team performance and innovation [20, 21]. However, team members have many different goals, ranging from personal-level goals such as “publish a high-impact paper to get promoted” to unit-level goals such as “secure enough funding to keep my lab running.” Team leaders must ensure that participants understand and buy into the high-level goal of the project (e.g., “to discover biomarkers”) and understand how their work contributes to the mission. The mission, vision, and goals should be clear, encompass the work of all participants, and be reiterated frequently. As the science evolves, the mission and goals may also evolve, so it is critical that they be revisited during a project.
*Impact on reproducibility:* Developing a shared mission, vision, and goals requires collaborators be objective about their own biases, intentional about how the work should be conducted, honest in the conversations about what they can truly achieve, and welcome the contributions of others. The development of a shared mission requires collaborators to think about how both work and credit are fairly allocated, and how the project contributes to the broader scientific community. By doing this early in the life of the team, collaborators develop a plan to build reproducibility in from the start.
2. Build a culture of trust, accountability, openness, inclusivity, and constant learning. Successful teams build a culture of psychological safety, “a shared belief held by members of a team that the team is safe for interpersonal risk taking” [22, 23]. In a psychologically safe environment, leadership listens to all voices, encouraging all team members to participate in a meaningful way. Team members learn to share failures, ask questions, and challenge the assumptions of others, in a respectful way that encourages open discussion and debate and discourages personal attacks. Team members meet their commitments and trust colleagues to do the same.
*Impact on reproducibility:* A culture of trust and openness increases the likelihood of discovering and sharing mistakes, disconnects, and misunderstandings, because all topics are open for discussion. Collaborators can, likewise, be open about the competing incentives driving their choices and behaviors. Cross training of trainees across labs, a coordination mechanism that has been associated with more successful outcomes [24], helps collaborators understand each other’s science and may also expose misunderstandings and identify confounding variables. This open environment encourages cross-disciplinary conversations, because everyone is a learner and everyone is an expert in some aspect of the project. Semi-independent testing of results is common in team-based projects that involve multiple labs conducting the same assay and can expose minute differences (e.g., ambient temperature in the lab, order of operations) that impact results.
3. Facilitate interdisciplinary conversations on approaches, methods, and results. Disciplinary diversity enhances innovation when diverse viewpoints are brought together for team problem-solving [25]. Discussion of research methods across disciplines includes an objective analysis of research approaches, their limitations, and shortcomings. Sharing methods across disciplines can spark innovative use of one field’s methods, adapted to another field. These conversations take time and energy, so teams must build that time both into the beginning of the project and along the way. Engaging a trained facilitator for interdisciplinary conversations may help, or teams can seek out tools such as the Toolbox Dialog Initiative [26], a process designed to support and enhance these conversations. Successful interdisciplinary conversations surface the varying perspectives each discipline brings, ensuring each contribution is treated as valuable.
*Impact on reproducibility:* Cross-disciplinary conversations can surface biases that influence study design. Development of shared protocols can also bring to light differences in approaches that may impact the methods reproducibility of the research. These interdisciplinary conversations provide another opportunity for the discovery of disconnects among collaborators. If collaborators are collecting common data, providing a forum for biostatisticians and data managers to share analysis code can both encourage efficiency through reuse and provide an opportunity to find mistakes or misconceptions early.
4. Build strong research support systems (information management, scientific coordination and project management, and communication systems). Strong research support systems can help the team focus on *how* the research gets done, making their vision and goals concrete and trackable, which leads to accountability, transparency, and a culture of openness from the beginning. It is critical that teams think not only about their data, but also about their information, such as how decisions were made, who is responsible for tasks, and when tasks are due. Communication systems, likewise, should be transparent but targeted, focused on getting the right information to the right team member at the right time. Additionally, coordinating research tasks can bring to light areas of synergy and of overlap where additional coordination is needed.
*Impact on reproducibility:* Coordination and communication systems have a dramatic impact on a team’s ability to achieve its scientific goals, in part because they so strongly influence the other best practices. Tracking decision-making processes and keeping accurate records (e.g., how were inclusion/exclusion criteria operationalized?) saves time and effort, allows for more accurate and complete reporting of both processes and results, and preserves the record for anyone wanting to reproduce the research.
5. Build accessible, transparent data management systems. Strong teams invest in data systems that are accessible to appropriate members of the team while allowing for overall transparency. Distributed teams should consider how team members will access secure drives, especially across institutions. Interdisciplinary teams must discuss what constitutes data in the various research fields, as well as how data and metadata will be organized. A robust data-sharing policy that anticipates the timely dissemination of useful, usable data sets can encourage careful data and methods documentation.
*Impact on reproducibility.* Accessible, transparent data management systems allow for robust, trustworthy data sharing within the project and with the scientific community. This data sharing requires both the scientists producing and those using the data to subject it to extra scrutiny. Research has shown that reusing someone else’s data requires ongoing, iterative conversations about the context in which data were collected, how the data set was produced from the raw data, what variables really mean, and any limitations of the data [27]. These conversations can surface errors in the data, in the analysis code, in the codebook, and even in the analyses. Records and quality management involve data retention, description of experiments (metadata), and retention of analytic methods (e.g., source code) ensures methods reproducibility. Adoption of minimum information about microarrays (MIAME) for reporting scientific results is an example of this approach.
6. Foster strong, functional leadership. Leadership has a critical, but understudied, impact on the success of Translational Teams [28]. Typically, teams are developed through a vertical leadership model that dynamically changes its roles depending on the team’s evolutionary phase [29]. In the “Development” and “Conceptualization” phases of Translational Team evolution (Fig. 1), leaders provide mentoring, coaching, and discipline integration, and establish the vision and goals. In the “Implementation” and “Translation” phases, leaders promote team behaviors by monitoring team performance, developing team capacity, providing feedback, and facilitating “sense making” [29]. In highly functioning teams, an emergent property is the demonstration of shared leadership where responsibility and accountability are shared among individuals in the team [29, 30]. Written authorship policies that accommodate team members’ diverse disciplinary practices clarify team operations and contribute to effective distributed accountability.
*Impact on reproducibility:* Best practices of leadership include fostering open communication to build trust and enhance transparency. Fostering accountability enhances the opportunity for independent checks on experiments, analysis, and conclusions. Effective project management capabilities facilitate shared goal achievement [31], which, too, strengthens the team’s trust in one another. Intentional leaders expect members to be trained and compliant with institutional requirements, and ensure checks and balances on experimental design and data interpretation. Modeling ethical behaviors provides examples for trainees. Mentors deeply involved in examining primary data reduce the opportunity for DRPs [32]. Monitoring the climate of the team to create an ethical workplace reduces unintended consequences of students being pressured to publish work prematurely [33].

### Implementation of SciTS Best Practices at CTSA Hubs

We encourage CTSA hub team-science experts to consider integrating trainings and evidence-based interventions around each of these six areas into their existing team-science offerings. While every team is different, our experience has shown that the two areas most challenging for teams are: 1. facilitating interdisciplinary conversations about approaches, methods, and results and 2. fostering strong, functional leadership. Most researchers simply have not received training in these two skill sets; however, these are two areas where the SciTS field and others have conducted substantial research and interventions or training programs are available. Busy researchers may not be intrinsically motivated to participate in such trainings and interventions. One potential incentive is to embed tailored team science training as a component of onboarding pilot projects or newly funded teams, but we must be careful to ensure we do not overburden such nascent teams with additional requirements. Ultimately, the most persuasive evidence is seeing their peers conducting high-impact, reproducible research.

### Building Team-Science Infrastructure and Innovative Practices at the UW

The RFA for the CTSA hubs includes a focus on supporting team science. At the University of Wisconsin (UW), we have focused our efforts in four key areas of team-science support: 1. Team-science education, 2. Team-science interventions, 3. Science of Team Science research, and 4. Development of a culture of team science. The goal of these efforts is to help Translational Teams conduct high-impact, rigorous, and reproducible science by building team-science infrastructure that leverages SciTS best practices. First, we are developing a robust set of modular courses, designed to meet the needs of researchers from graduate students through senior researchers. Second, we are developing interventions for both nascent and experienced teams. One such intervention is focused on Collaboration Planning. Adapted from work by SciTS researchers at the National Cancer Institute and National Science Foundation, this 90-minute facilitated session guides teams through discussions on 10 areas of focus, including the overarching goals of the project, information and data management plans, and authorship policies. We have conducted sessions with 20 teams; all have found it surfaces issues teams had not previously discussed, such as authorship policies, communications management, and conflict resolution procedures [34]. Third, our team-science activities have rigorous evaluation and research components to ensure that the evidence-based services we deliver contribute to the success of UW teams and can be disseminated to other CTSA hubs and beyond. Finally, we are actively engaged in enhancing the culture of team science at the UW by developing services to support teams (e.g., helping write team-science components of grant proposals) and working with UW tenure and promotion committees to incorporate policy language to clarify support for team science. Because each CTSA has crafted its own approach to supporting team science, the network has the potential to learn what approaches do or do not translate to improvements in reproducible research.

### Assessing Antecedents for Reproducible Science

A major opportunity for the field of clinical and translational science is to identify example behaviors that are antecedents of and consistently foster reproducibility-enhancing behaviors. Building on the SciTS literature, we propose a rubric for assessing those behaviors (Table 1). For each best practice, the specific behavior and examples of high and low performing teams are listed. For example, in the practice of building a culture of trust, a high-performance team will see that team members feel comfortable questioning each other’s data and conclusions, while a low-performing team will have team members that get defensive about being questioned and rarely question others. Measurement of these behavioral antecedents will enable inferences to be drawn about the culture of reproducibility in a team and evaluation of impact of team science training on good research practices. Team-science experts at CTSA hubs can help Translational Teams adapt this rubric to their local team context, which includes the institutional norms and culture that might run counter to team-based reproducibility behaviors. The strength of the team-based practices to encourage reproducibility behaviors is that team members hold one another accountable and work together to create a culture of reproducibility. We believe this works to mitigate the negative impact of an institutional culture focused exclusively on tenure and promotion, especially given that the behaviors described here actually contribute to higher impact, more reproducible science. Team-science experts can also help the team target their efforts (i.e., areas closer to “low performance” than “high performance”), allowing the team to focus its limited resources on those areas. We hope to explore the use and impact of this rubric in future research, including investigating questions such as which of the six areas most greatly predict overall team performance.

**Table 1.** Rubric for assessing the conduct of reproducible science in a Translational Team

Best practice in team science	Specific behavior	High performance	Low performance (DRPs)
Mission/Vision/Goals	Build consensus around the mission, vision, and goal, and ensure individuals goals are also met	Team members articulate the same mission, vision, goals.	Individuals have different goals for the teams and engage in behaviors that do not serve the larger goals.
Culture of trust	Create a culture of psychological safety	Team members feel comfortable questioning each other’s data and conclusions.	Team members are defensive about being questioned and rarely question others.
Interdisciplinary conversations on approaches, methods, and results	Include disciplinary diversity	Statisticians/bioinformatics represented at early design stage.	Analysis expertise involved post hoc.
	Define hypotheses for testing	Primary endpoint established and reported. Study appropriately powered.	Testing post hoc, endpoints selected from multiple options that meet prior bias or statistical significance.
	Discuss methods, analysis, and results	Hypothesis testing.	Primary endpoint established and reported. Study appropriately powered.
	Share experimental design/description	Experimental details are transparent, discussed at team meetings, methods, and conclusions challenged.	Team scientists do not know the details of other team members’ experiments
	Conduct semi-independent replication	Subgroups within Translational Team conduct similar experiments.	Experiments not replicated; published as n = 1.
Research support systems	Conduct team business openly and transparently	Collaboration plan developed by team – agreed upon and used.	Lack of consistency and transparency in communication and operations.
Data management	Build a data management system that works for all team members with appropriate data structure, access, and archival	Data system to archive experimental results, securely accessible to all team members.	Experiments kept in individual lab archives not accessible to other team members.
	Build robust data analysis pipelines	Source code, metadata archived with primary data.	Data analysis pipeline not shared/available.
Leadership	Attribute authorship and IP responsibly	Authorship, IP policies exist and are based on explicit contribution criteria.	No written authorship policy. Authorship given to funders without direct study involvement; or to high-profile scientist in the field to enhance impact. Manuscript sections recycled.
	Foster a culture of mentoring	Co-mentoring and career development embedded in team development activities.	Scientists/students used as technical support.
	Require and track training above and beyond institutional or funder requirements	Team members current with compliance, responsible conduct of research (RCR), and security. Security/privacy/ethical issues incorporated into team discussions.	Training, compliance, and RCR not visible or consistent across team members.

DRP, detrimental research practices.

### Challenges to Team-Based Reproducibility Interventions

The potential of Translational Teams to serve as at least a partial solution to the problem of reproducibility rests, in part, on current norms and approaches. As the conduct of clinical and translational science evolves over time, the way Translational Teams work may also change and evolve. Important trends in research that have implications for reproducible science include the development of new methodologies, data science, and globalization of interprofessional research. New data-intensive, next-generation methodologies are not always understood by all team members, which reduces the ability of the team to understand, at a deep level, certain experimental results. Team leaders need to be sensitive to this problem, encouraging the training of team members to understand new methods as they are adopted by the team. Advances in data science lead to problems in reproducible computing, where data processing and analysis platforms can be “hidden,” preventing reproducible replication. Finally, widely distributed (globalized) teams face unique challenges in asynchronous communication. Effective data and project management are required in these cases.

## Conclusions

Enhancing the reproducibility of preclinical research is vital to the success of the CTSA consortium. Detrimental Research Practices emerge from complex incentives and rewards within the research environment, and existing solutions have fallen short. We propose integration of evidence-based practices from the SciTS field that reinforce reproducibility practices throughout the interprofessional team lifecycle. Best practices in team science ensure research occurs in a visible, transparent, and incentive-aligned manner, ensuring that experiments can be described, analytical methods replicated, and results extensible for building more robust clinical interventions. Expanding the range of behavioral antecedents to operationalize reproducible team science will advance the translational goals of the CTSA program.
